# p38γ and p38δ Mitogen Activated Protein Kinases (MAPKs), New Stars in the MAPK Galaxy

**DOI:** 10.3389/fcell.2016.00031

**Published:** 2016-04-14

**Authors:** Alejandra Escós, Ana Risco, Dayanira Alsina-Beauchamp, Ana Cuenda

**Affiliations:** Department of Immunology and Oncology, Centro Nacional de Biotecnología, Spanish National Research Council (CSIC)Madrid, Spain

**Keywords:** p38γ, p38δ, inflammation, innate response

## Abstract

The protein kinases p38γ and p38δ belong to the p38 mitogen-activated protein kinase (MAPK) family. p38MAPK signaling controls many cellular processes and is one of the most conserved mechanisms in eukaryotes for the cellular response to environmental stress and inflammation. Although p38γ and p38δ are widely expressed, it is likely that they perform specific functions in different tissues. Their involvement in human pathologies such as inflammation-related diseases or cancer is starting to be uncovered. In this article we give a general overview and highlight recent advances made in defining the functions of p38γ and p38δ, focusing in innate immunity and inflammation. We consider the potential of the pharmacological targeting of MAPK pathways to treat autoimmune and inflammatory diseases and cancer.

## Introduction

The activation of p38 Mitogen Activated Protein Kinases (p38MAPKs) is one of the main signal transduction mechanisms by which the cell adapts to changes in the environment. There are four p38MAPK isoforms in mammalian cells encoded by different genes: p38α (*MAPK14*), p38β (*MAPK 11*), p38γ (*MAPK 12*), and p38δ (*MAPK 13*) (Cuenda and Rousseau, 2007). p38α was the first p38MAPK family member identified, therefore the most studied and best-characterized isoform; most of the literature on p38MAPK refers to p38α.

The four p38MAPK isoforms are widely expressed, but their expression pattern varies in tissues. p38α is ubiquitously expressed in all cell types and tissues, although expression levels are lower in the brain, liver, and pancreas than in other tissues. p38β is highly expressed in the brain, thymus, and spleen; its expression is lower in the adrenals, lung, kidney, liver, pancreas, and heart, and it is not expressed in skeletal muscle (Beardmore et al., 2005). p38γ is very abundant in skeletal muscle, although its expression in most other tissues is lower (Mertens et al., 1996; Beardmore et al., 2005). p38δ levels are high in pancreas, intestine, adrenal gland, kidney, and heart (Goedert et al., 1997; Jiang et al., 1997; Beardmore et al., 2005).

**Figure 1:**
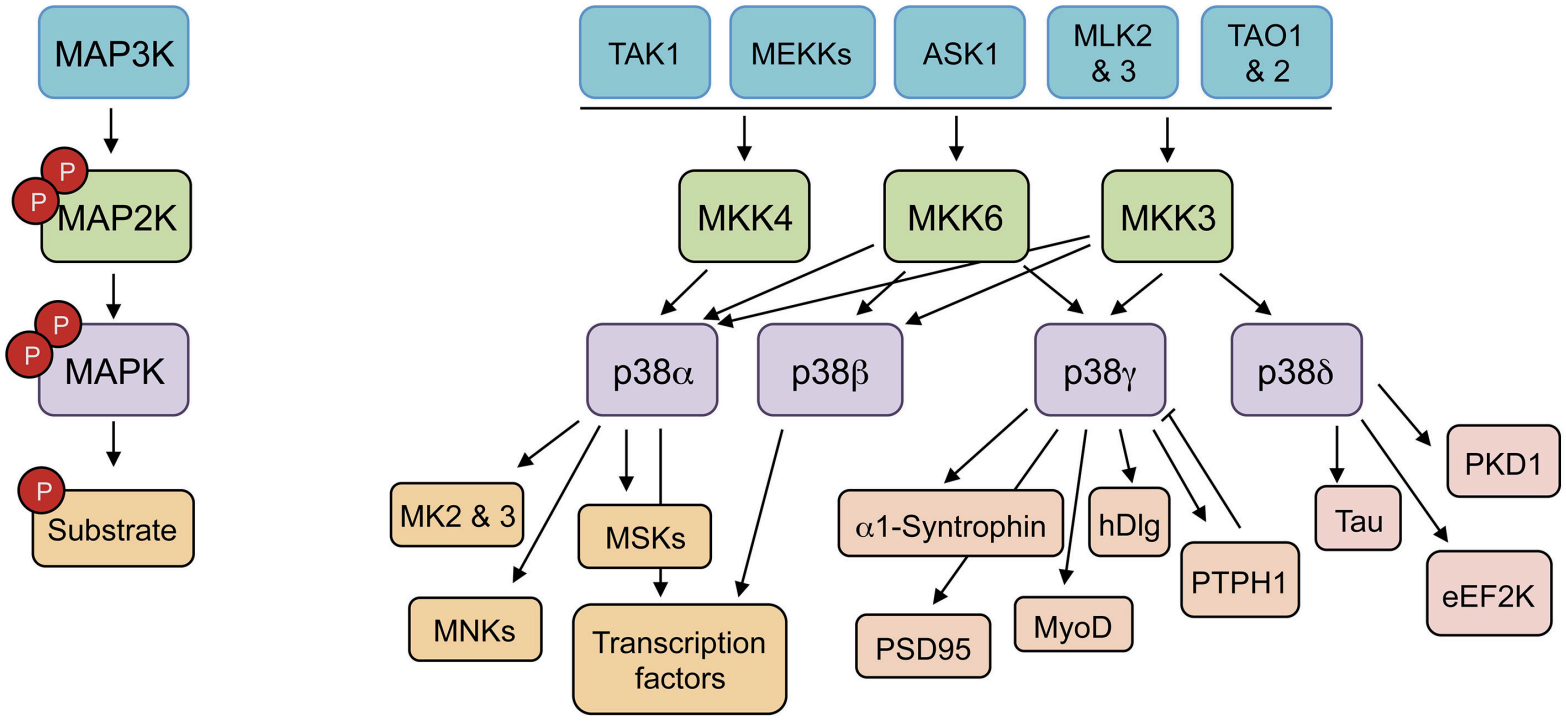
**p38MAPK pathways consist of several MAPK kinase kinases (MAP3K), three MAPK kinase (MAP2K), and four p38MAPK**. These pathways can be activated by many stimuli, including growth factors, inflammatory cytokines, and a wide range of cellular stresses. The p38MAPKs phosphorylate different substrates, including protein kinases, cytosolic substrates, and transcription factors. ASK1 (apoptosis signal-regulating kinase-1), eEF2K (eukaryotic elongation factor 2 kinase), hDlg (human disc large), MEKK (MAPK/ERK kinase kinases), MK (MAPK-activated protein kinase), MLKs (mixed-lineage kinases), MNK (MAPK-interacting protein kinase), MSK (mitogen and stress-activated kinase), PKD1 (protein kinase D1), PSD95 (post-synapse density 95), PTPH1 (protein tyrosine phosphatase H1), TAK1 (TGF β-activated kinase 1), TAO (thousand-and-one amino acid).

p38MAPKs are strongly activated by a wide variety of environmental and cellular stresses or by inflammatory cytokines, but are poorly activated by serum or growth factors (Cuenda and Rousseau, 2007). All p38MAPKs are Serine/Threonine kinases that catalyze the reversible phosphorylation of proteins. They are activated by dual phosphorylation of the TGY activation motif mediated by the MAPK kinases (MAP2K) MKK3, and MKK6, and in the case of p38α also by MKK4 (Remy et al., 2010). The activation of distinct p38MAPK isoforms is regulated by the selective and synchronized action of two kinases, MKK3 and MKK6. These two MAP2Ks are implicated in p38α, p38γ, and p38β activation in response to general environmental stresses in mouse embryonic fibroblasts (Remy et al., 2010). However, MKK3 is the major kinase responsible for p38δ activation (Remy et al., 2010). MKK3 and MKK6 are in turn activated upon phosphorylation of Serine/Threonine residues by a MAPK kinase kinase (MAP3K). Several MAP3Ks, including MAPK/ERK kinase kinases (MEKK), TAO1 and 2, ASK1 (apoptosis signal-regulating kinase-1), MLKs (mixed-lineage kinases), and TAK1 (TGF β-activated kinase 1) activate p38MAPK cascade; the specific MAP3K that is required appears to be stimulus and cell type specific (Cuenda and Rousseau, 2007) (Figure 1).

p38MAPK family can be further divided into two subsets, p38α/p38β and p38γ/p38δ, based on sequence homology, substrate specificities, and sensitivity to chemical inhibitors. In this review, we provide an overview of p38γ and p38δ (also called alternative p38MAPKs), which play important roles in the innate immune response, in inflammation and inflammation-related diseases such as cancer or arthritis.

## General features of p38γ and p38δ signaling pathways

One of the hallmarks used for the division of p38MAPK into two subgroups is the protein sequence similarity. p38γ and p38δ sequences are highly similar to each other (~70% identity), whereas p38α and p38β display higher similarity (75% identity). In contrast, p38γ and p38δ are more divergent in sequence to p38α (~60% identical to p38α) (Risco and Cuenda, 2012). These two p38MAPK subgroups also differ on their sensitivity to kinase inhibitors. Pharmacological experiments demonstrated that only p38α and p38β are inhibited by certain compounds, which are ATP competitors, such as SB203580 and other pyridinyl imidazoles, whereas p38γ and p38δ are not affected by these drugs (Goedert et al., 1997; Kuma et al., 2005; Bain et al., 2007). However, the diaryl urea compound BIRB796, a molecule that functions allosterically and is a potent inhibitor of p38α and p38β, also inhibits p38γ and p38δ at higher concentrations in cell-based assays. This compound has provided a good tool for identifying physiological substrates and roles of the alternative p38MAPK isoforms (Kuma et al., 2005; Cuenda and Rousseau, 2007; Risco and Cuenda, 2012). Nonetheless, due to the lack of specific p38γ and p38δ inhibitors, the information about the physiological substrates and the biological function of these kinases is limited compared to the extensive knowledge of p38α functions.

The use of kinase inhibitors and the genetic deletion of specific p38MAPK isoforms has showed that they have some overlapping substrates and functional redundancy; however, there are particular proteins that are better substrates for p38α/p38β than for p38γ/p38δ and the other way around (Kuma et al., 2005; Cuenda and Rousseau, 2007; Risco and Cuenda, 2012). Even more, protein kinases such as MAPK-activated protein kinase 2 (MK2) or MK3 are good substrates for p38α and p38β, but cannot be phosphorylated by other p38MAPK isoforms (Cuenda et al., 1997; Goedert et al., 1997; Cuenda and Rousseau, 2007; Arthur and Ley, 2013) (Figure 1).

It has been reported that p38δ kinase phosphorylates the neuronal microtubule-associated protein Tau (Feijoo et al., 2005), the eukaryotic elongation factor 2 kinase (eEF2K) (Knebel et al., 2001), the protein kinase D1 (PKD1) (Sumara et al., 2009), which controls insulin exocytosis in pancreatic beta cells and chemotaxis in neutrophils, and the signal adaptor p62, which controls mTORC1 activation, autophagy, and tumor growth (Linares et al., 2015).

Several physiological substrates for p38γ have been described taking advantage of a feature that makes p38γ unique among other MAPKs. p38γ possesses a short C-terminal sequence (-KETXL), which binds to PDZ domains. p38γ associates with PDZ-domain containing proteins, such as α1-syntrophin, SAP (synapse-associated protein) 90/PSD (post-synapse density) 95, hDlg (human disc large also known as SAP97) and the protein tyrosine phosphatase PTPH1 and under stress conditions it is able to phosphorylate them and modulate their activity (Hasegawa et al., 1999; Sabio et al., 2004, 2005; Hou et al., 2010). For example, changes in the osmolarity of the environment trigger p38γ activation in the cytoplasm, which phosphorylates hDlg. Phosphorylation of hDlg leads to its dissociation from the cytoskeletal guanylate kinase-associated protein (GKAP) and therefore from the cytoskeleton (Hasegawa et al., 1999; Sabio et al., 2004, 2005; Hou et al., 2010). In addition, the interaction of p38γ with the single PDZ domain of PTPH1 enables this phosphatase to dephosphorylate p38γ, but not p38α, *in vitro* and in cells over-expressing both proteins (Hou et al., 2010; Chen et al., 2014). So far, the only physiological p38γ substrate that does not require PDZ domain binding interactions is the transcription factor MyoD, whose phosphorylation by p38γ results in a decrease in its transcriptional activity (Gillespie et al., 2009).

p38MAPKs act normally by direct phosphorylation of substrates on Serine or Threonine residues followed by Proline, however, there are some examples showing that p38α and also p38γ may also have kinase independent roles by associating to protein targets and modulating their function in the absence of phosphorylation (reviewed in Cuadrado and Nebreda, 2010; Risco and Cuenda, 2012). For example, it has been shown that p38γ regulates nuclear protein complexes independently of its kinase activity. Changes in the osmolarity cause the accumulation of p38γ in the nucleus where it interacts with nuclear hDlg. In the nucleus, hDlg forms a complex with the proteins polypyrimidine tract-binding (PTB) protein-associated splicing factor (PSF) and p54nrb, and with various RNAs. p38γ regulates hDlg-PSF complex dissociation independently of hDlg phosphorylation by displacing PSF from hDlg, since both proteins, p38γ and PSF, bind to PDZ1 domain of hDlg. This has been shown comparing cells from knockin mice expressing an endogenous kinase-inactive p38γ mutant with cells from mice lacking p38γ (Sabio et al., 2005, 2010; Remy et al., 2010; Risco and Cuenda, 2012). The studies on p38γ-hDlg-GKAP and p38γ-hDlg-PSF protein complexes indicate that, through its ability to shuttle between cytoplasm and nucleus, p38γ might provide a connection between two processes critical for adaptation to environmental changes: gene expression and cytoskeletal reorganization.

## Some physiological roles of p38γ and p38δ MAPK pathways

**Figure 2:**
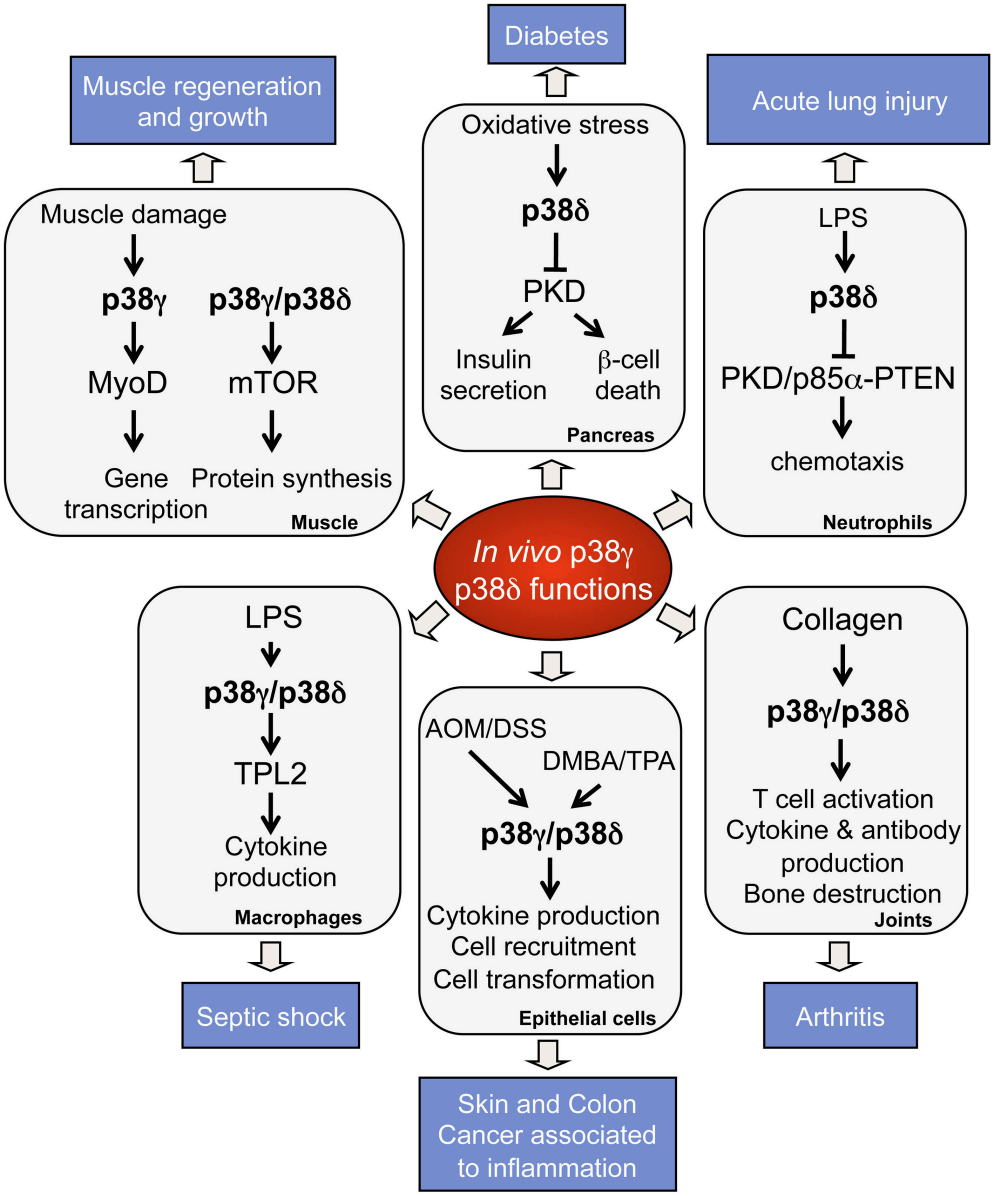
**Physiological roles and pathological implications of p38γ and p38δ**. p38γ and p38δ are key players in the regulation of many biological functions, which contribute to physiological processes. Deregulation of p38γ and p38δ leads to the development of several pathological conditions.

Studies using knock-out mice have provided important information concerning p38γ and p38δ functions *in vivo* and in pathological conditions (Figure 2). p38γ and p38δ deficient mice are viable and have not apparent phenotypes (Sabio et al., 2005, 2010; Remy et al., 2010; Risco and Cuenda, 2012). Nonetheless, there are reports showing the implication of p38γ and p38δ in tissue regeneration, cancer, and metabolic diseases (Sabio et al., 2005, 2010; Remy et al., 2010; Risco and Cuenda, 2012). Thus, it has been described that p38δ regulates insulin secretion and pancreatic β cells death implying a central role in diabetes (Cuenda and Nebreda, 2009; Sumara et al., 2009). p38δ is also crucial in neutrophil chemotaxis pathway, contributing to acute respiratory distress syndrome (ARDS) (Ittner et al., 2012), and in mediating IL-13-driven mucus overproduction in human airway epithelial cells in chronic inflammatory lung diseases (Alevy et al., 2012).

Since p38γ expression is very high in skeletal muscle and its expression is induced during muscle differentiation (Cuenda and Cohen, 1999; Tortorella et al., 2003; Perdiguero et al., 2007), it is not surprising that it plays a fundamental role in this process. Thus, p38γ knockdown impairs cardiomyocyte formation (Ramachandra et al., 2016) and p38γ and p38δ promote cardiac hypertrophy by modulating the mTOR pathway (González-Terán et al., 2016). Moreover, studies in p38γ deficient mice reported that p38γ plays a pivotal role in blocking the premature differentiation of skeletal muscle stem cells, the satellite cells that participate in adult muscle regeneration (Gillespie et al., 2009). Also, p38γ is required for the upregulation of PGC-1α [peroxisome proliferator-activated receptor-γ (PPARγ) coactivator-1α] in mitochondrial biogenesis and angiogenesis in response to endurance exercise in mice, which is critical for skeletal muscle adaptation (Pogozelski et al., 2009).

In addition, p38γ and p38δ are involved in the modulation of some processes implicated in cellular malignant transformation, such as proliferation, cell cycle progression, apoptosis, or cell migration. Using mouse embryonic fibroblasts derived from mice lacking p38γ or p38δ, it has been shown that deletion of either p38γ or p38δ increases cell migration and metalloproteinase-2 secretion, whereas only p38δ deficiency impairs cell contact inhibition. Also, lack of p38γ in K-Ras-transformed fibroblasts leads to increased cell proliferation as well as tumorigenesis both *in vitro* and *in vivo* (Cerezo-Guisado et al., 2011). These pieces of evidence indicates that p38γ and p38δ have a role in tumor suppression; however, there are other studies reporting a pro-oncogenic function for these kinases, for example in the development of breast and skin cancer (reviewed in Risco and Cuenda, 2012).

## p38γ and p38δ in the innate response and in inflammation

The use of genetically modified mice lacking one or more p38 isoform has provided strong evidence of the p38γ and p38δ importance in the innate immune response and in inflammation (Figure 2). The innate immune system is the front line of defense against invading pathogens, and uses evolutionarily conserved high-affinity receptors (pattern recognition receptors, PRRs) that recognize specific constituents of bacteria or virus, endogenous cytokines and host cell-derived components (Kawai and Akira, 2007). The activation of PRRs in the innate immune cells leads to secretion of inflammatory cytokines and other mediators, which induce an inflammatory response. This inflammatory response promotes the recruitment of additional immune cells, the elimination of infectious agents, and the induction of tissue repair (Kawai and Akira, 2007). The production of cytokines during the innate response is also important for the subsequent induction of the adaptive immune response (Iwasaki and Medzhitov, 2010). The stimulation of all PRRs by pathogen-associated molecules activates both MAPKs and NFκB pathways, which are crucial to generate immune responses (Cuenda and Rousseau, 2007; Gaestel et al., 2009; Arthur and Ley, 2013).

The important role of the MAPK p38α in the innate immune response and in inflammation has been uncovered mainly by studies using a range of p38α inhibitors or the constitutive deletion of its physiological substrates, or activators (Cuenda and Rousseau, 2007; Gaestel et al., 2009; Arthur and Ley, 2013). Much less is known about the importance of p38γ and p38δ in these processes. However, recent studies show that in macrophages and dendritic cells (DC), which are key mediators of the inflammatory response, the deletion of both p38γ and p38δ impaired the innate immune response to lipopolysaccharide (LPS), a Toll-like receptor 4 (TLR4) ligand (Risco et al., 2012). In these cells p38γ and p38δ are necessary to maintain steady-state levels of TPL-2, the MKK kinase that mediates ERK1/2 activation in response to TLR stimulation (Gantke et al., 2011). ERK1/2 are other MAPK family members that play a central role in cytokine production. p38γ and p38δ deficient macrophages (p38γ/δ^−/−^) showed substantially lower levels of TPL2 protein, and therefore lower MKK1-ERK1/2 activation and inflammatory cytokine production (Risco et al., 2012). Exogenous expression of TPL-2 in p38γ/δ^−/−^ macrophages not only increased ERK1/2 activation, but also rescued TPL-2-dependent TNFα production in response to LPS (Risco et al., 2012).

p38γ and p38δ signaling has complex pro- and anti-inflammatory effects on cytokine production in innate immune responses. Production of TNFα, IL-1β, and IL-10 is severely reduced in LPS-stimulated macrophages from p38γ/δ-deficient mice, whereas IL-12 and IFNβ production increases (Risco et al., 2012). p38γ and p38δ regulate IL-1β and IL-10 production at the transcriptional level, whereas regulation of TNFα is at the secretion level in bone marrow derived macrophages stimulated with LPS (Risco et al., 2012). In LPS-stimulated liver macrophages, p38γ and p38δ are required for the translation of *Tnf* mRNA through inhibitory phosphorylation of eEF2K that leads to activation of eEF2 (González-Teran et al., 2013). Furthermore, in TPA-stimulated keratinocytes, p38γ and p38δ are required for IL-6, IL-1β, and CXCL1 transcription (Zur et al., 2015). The exact mechanisms by which p38γ and p38δ regulate the production of cytokines and chemokines in different cells are still largely unknown and further studies are needed to determine them.

Using p38δ deficient mice it has been shown that this p38MAPK isoform is important in neutrophils migration and in their recruitment into inflammatory sites in lung (Ittner et al., 2012). The degree of inflammation and the associated organ damage is a consequence of complex pro- and anti-inflammatory responses, which involve the regulation of neutrophil recruitment and migration in a cell-autonomous manner. p38δ-deficient neutrophils show a defect in chemotaxis, which is caused by increased activity of the p38δ substrate, the kinase PKD1 (Sumara et al., 2009). PKD1 phosphorylates p85α to enhance its interaction with PTEN, leading to increased PTEN activity and lower cell migration (Ittner et al., 2012). Appropriate signaling in neutrophils is essential to resolve inflammation without causing inappropriate organ damage.

Overall, these data strongly suggest that p38γ and p38δ have a key role in the mechanisms leading to inflammation.

## p38γ and p38δ in inflammatory diseases

The role of p38γ and p38δ in inflammation *in vivo* is further supported by experiments in other mouse animal models. The reaction to bacterial LPS is a well-characterized innate immune response that leads to endotoxic or septic shock, due primarily to TNFα overproduction. Thus, p38γ/δ-deficient mice are less sensitive to endotoxic shock than wild type mice following LPS challenge and this is associated with a decrease in serum levels of inflammatory cytokines such as TNFα, IL-1β, or IL-10 (Risco et al., 2012). The acute liver failure caused by LPS is also suppressed in mice that lack p38γ and p38δ in myeloid cells (González-Teran et al., 2013). In addition, p38δ deletion results in decreased alveolar neutrophil accumulation, reduces acute lung inflammation, and protect from acute lung injury (ALI) induced by LPS (Ittner et al., 2012). Also, there are evidence that p38δ mediates mucus production in chronic inflammatory lung disease, since either the knockdown or inhibition of p38δ, but not of p38α, can block inflammatory IL13-induced mucus production in human airway epithelial cells (Alevy et al., 2012).

The role of p38γ and p38δ isoforms in other inflammatory diseases such as arthritis has recently been shown in a collagen-induced arthritis (CIA) mouse model. Combined p38γ and p38δ deficiency markedly reduced arthritis severity by suppressing clinical disease and bone destruction, compared with that in wild type mice (Criado et al., 2014). p38γ/δ deficient mice have lower mRNA expression of IL-17 and IFNγ in joints, and lower levels of pathogenic anti-collagen antibodies, IL-1β, and TNF-α in the serum than wild type mice (Criado et al., 2014). p38γ and p38δ also seem to control T cell activation, for example lymph node T cells from p38γ/δ-deficient mice show reduced proliferation and interferon (IFN)γ and IL-17 production (Criado et al., 2014). Moreover, p38γ/δ deficient mice showed a lower Th17 cell frequency and a greater Treg/Th17 ratio, both of which are linked to successful therapy in rheumatoid arthritis. The crucial role of p38γ/δ in synovial inflammation, bone erosion, as well as cytokine production suggests that they could serve as targets of therapy in rheumatoid arthritis as an alternative to traditional p38α inhibitors, which have proven minimally effective in human disease (Gaestel et al., 2009; Arthur and Ley, 2013).

During the last few years the role of p38γ and p38δ in cancer associated with chronic inflammation has been studied. Chronic inflammation is linked with an increase in malignant disease. Almost 20% of human cancers are related to chronic inflammation caused by infections, exposure to irritants or autoimmune diseases (Hanahan and Weinberg, 2011; Crusz and Balkwill, 2015). Colitis-associated cancer (CAC) is a colon cancer subtype associated with inflammatory bowel disease, such as that occurring in ulcerative colitis or Crohn's disease. Using the azoxymethane (AOM)/dextran sodium sulfate (DSS) mouse model of CAC it has been shown that p38γ and p38δ have a pro-oncogenic role by regulating inflammatory signaling to promote colon tumorigenesis, thus linking inflammation and cancer in CAC (Del Reino et al., 2014). Mice deficient in p38γ and p38δ display a decrease in cytokines and chemokines production and in inflammatory cell infiltration in the colon of treated animal and produce fewer colon tumors than control mice (Del Reino et al., 2014). p38γ and p38δ in hematopoietic cells are important for CAC development. Lethally irradiated wild type mice reconstituted with bone marrow from p38γ/δ-null mice exhibited less tumor formation, cytokine production, and immune cell infiltration, whereas p38γ/δ-deficient mice reconstituted with wild type bone marrow showed more tumor formation, cytokine production, and immune cell infiltration than controls (Del Reino et al., 2014). The pro-oncogenic role of p38γ and p38δ was also confirmed in the two-step 7,12-dimethylbenz(*a*)anthracene (DMBA)/12-*O*-tetradecanoylphorbol-13-acetate (TPA) chemical skin carcinogenesis model (Schindler et al., 2009; Zur et al., 2015). p38γ/δ-deficient mice showed diminished cytokine production and are resistant to tumorigenesis (Zur et al., 2015). Overall, all of these results suggest potential therapeutic prospects by targeting p38γ and p38δ for treatment of cancer. Future studies examining the effects of cell type-selective p38γ and p38δ targeting at different stages of carcinogenesis will elucidate the functional roles of these two alternative p38MAPKs in the tumorigenesis process, and will guide future therapeutic strategies.

## Conclusion and perspective

The role of p38γ and p38δ has been some times ignored since most of the studies to date have focused on p38α, which is the most abundant p38MAPK isoform. Nonetheless, in the last years significant progress in understanding the functions of p38γ and p38δ *in vivo* has been achieved. It is now clear that p38γ and p38δ are crucial in innate response, inflammation, and inflammatory diseases. Therefore, they deserve to be studied in greater depth as they represent pharmacological target for the development of drugs that might be useful for the treatment of inflammatory pathologies. In fact, development of more specific p38δ inhibitors has been shown to reduce mucus production in human airway epithelial cells (Alevy et al., 2012). However, the molecular mechanisms of how p38γ and p38δ regulate innate response, including the significance of the regulation of other signaling pathways components, cytokine production and the recruitment of immune cells, remain to be fully established. A better mechanistic understanding of p38γ/p38δ-regulated innate response will permit the design of p38γ and p38δ-based therapies, alternative to traditional p38α inhibitors, which have proven minimally effective in human inflammatory diseases (Gaestel et al., 2009; Arthur and Ley, 2013). The prospective that basic research on p38γ and p38δ could be translated to the treatment of human disease provides an exciting goal for future studies in the field.

## Author contributions

AC wrote the manuscript. AE, AR, DA made substantial contributions to conception and design, and acquisition of information. All authors contributor to the revision of the manuscript and approved the final version.

### Conflict of interest statement

The authors declare that the research was conducted in the absence of any commercial or financial relationships that could be construed as a potential conflict of interest.
